# Association of sugar-sweetened drinks with caries in 10- and 15-year-olds

**DOI:** 10.1186/s12903-020-01068-9

**Published:** 2020-03-19

**Authors:** Vinay Pitchika, Marie Standl, Carla Harris, Elisabeth Thiering, Reinhard Hickel, Joachim Heinrich, Jan Kühnisch

**Affiliations:** 1 Department of Operative Dentistry and Periodontology, School of Dentistry, Ludwig-Maximilians-Universität München, Goethestraße70, 80336 Munich, Germany; 2 Unit of Periodontology, Department of Restorative Dentistry, Periodontology, and Endodontology, University Medicine Greifswald, University of Greifswald, Greifswald, Germany; 3 Helmholtz Zentrum München - German Research Centre for Environmental Health, Institute of Epidemiology, Neuherberg, Germany; 4 Dr. von Hauner Children’s Hospital, University Hospital, LMU Munich, Munich, Germany; 5 Institute and Outpatient Clinic for Occupational, Social and Environmental Medicine, University Hospital of Munich, Ludwig-Maximilians-Universität München, Munich, Germany; 6 Allergy and Lung Health Unit, Melbourne School of Population and Global Health, The University of Melbourne, Melbourne, Australia

**Keywords:** Sugar-sweetened drinks, Caries, DMF index, Non-cavitated carious lesions, Epidemiology, Nutrition, Children

## Abstract

**Background:**

Sugar-sweetened drinks (SSDs) are known to be cariogenic, but this association has not been well investigated in population-based repeated cross-sectional studies in recent years. Therefore, this study examined whether SSD intake is associated with higher caries experience in 10- and 15-year-olds.

**Methods:**

The study sample included participants from the Munich study centre of two birth cohorts with data on non-cavitated caries lesions (NCCL/S), caries experience (DMF/S index), overall caries burden (DMF + NCCL/S) and SSD intake. In total, 915 and 996 children were included from the 10- and 15-year follow-ups, respectively. Intake (g/day) of SSDs, comprising cola, lemonade, ice-tea, sport/energy drinks, fruit squashes and nectars, was calculated from food frequency questionnaires. For analyses, the SSD intake was converted into portions (250 ml/day). Multiple logistic regression and prospective analysis models were performed to test associations between SSD intake and various definitions of caries, adjusting for sex, parental education, body mass index (BMI) categories, study cohort, plaque-affected sextants, mode of SSD consumption, energy content of SSDs, and total energy intake.

**Results:**

The mean overall caries burden at 10 and 15 years of age was 1.81 (SD: 2.71) and 6.04 (SD: 8.13), respectively. The average consumption of SSDs at the 10- and 15-year follow-ups was 0.48 (SD: 0.85) and 0.83 (SD 1.40) portions/day, respectively. After adjusting for confounders, in 10-year-olds, SSD intake was significantly associated with higher caries experience based on the indices DMF/S (adjusted odds ratio: 1.29; 95% CI: 1.06–1.57), NCCL/S (1.24; 1.03–1.49) and DMF + NCCL/S (1.27; 1.05–1.55). At the 15-year follow-up, SSD consumption was significantly associated with increased DMF/S index (1.12; 1.01–1.25) only. Prospective model associating 10-year SSD intake with 15-year caries experience was not significant.

**Conclusions:**

SSD intake significantly increases the caries burden in 10-year-olds, with attenuated effects in 15-year-olds. To prevent caries, SSD consumption should be reduced, especially in children and adolescents.

## Background

Dental caries remain among the most prevalent oral diseases in children and adolescents [1]. The aetiology of caries is multi-factorial [2]; among these factors, sugar intake is associated with an increased risk of caries [3, 4]. Fermentable dietary sugars constitute bacteria’s primary energy source, which then produce acids, such as lactic acid, which lowers the pH and leads to demineralization of the dental hard tissue [2]. Therefore, a diet rich in mono- and disaccharides might be a relevant caries risk factor. Previously, it was believed that sticky foods such as chocolates, cookies and cakes have a strong tendency of getting lodged into the pits and fissures of the teeth, thus increasing the risk of the development of caries [5]; however, the influence of sugar in liquid form might be increasingly impactful and has thus far not been fully considered. Along with current lifestyle changes, the consumption of sugar-sweetened drinks (SSDs) seems to have increased [6–8]. In addition to the excessive sugar load, these beverages are acidic in nature, which instantly reduces the oral pH. Although this effect can be initially neutralized by the buffering capacity of saliva [9], caries development may increase with greater frequency and extent of SSD exposure. However, it could be observed that individuals, particularly growing children, are replacing drinking water with SSDs. Such long-term exposure to SSDs is detrimental to dental health and poses a potential risk of the development of caries. Although clinical studies suggest a causal relationship between SSDs and caries, confirmatory evidence through population-based studies is scarce [10]. Some larger studies have been performed in children of younger ages [11, 12], but longitudinal evidence from population-based studies in adolescents is rare. Adolescence is a life stage during which dietary and other lifestyle changes begin to appear [13]. Therefore, this study aimed to analyse the association of SSD consumption on the caries experience of adolescents at 10- and 15-year follow-ups, adjusting for the relevant confounders. The tested null hypothesis was that SSD intake does not affect caries experience at both follow-up time points.

## Methods

The study protocols of the two birth cohorts were approved by the regional ethics committee, and written consent for the physical and dental examinations was obtained from all participating children and their guardians. The recommendations of the Strengthening the Reporting of Observational Studies in Epidemiology (STROBE) guidelines for observational studies were applied for reporting [14].

### Study population

The study sample was derived from two German prospective birth cohorts German Infant Nutritional Intervention programme plus (GINIplus) and Lifestyle-related factors, Immune System and the development of Allergies in East and West Germany plus (LISAplus). The present study used a cross-sectional design performed at two time points, when the same participants were followed-up at 10 and 15 years of age. Children born in the Munich centre who had available dental examination data and dietary information were included in this study. Details concerning the study’s background, representative recruitment strategy, inclusion and exclusion criteria have been described elsewhere [15–17]. In this study, a total of 915 (GINIplus: 557; LISAplus: 358) and 996 (GINIplus: 652; LISAplus: 344) participants were included from the 10- and 15-year follow-ups, respectively.

### Questionnaire

Before examinations at the study centre, a structured questionnaire and a food frequency questionnaire (FFQ) were sent to the subjects through post. Information on food intake over the past 12 months was obtained at each follow-up. The FFQs were filled predominantly by the parents at 10-year follow-up, and by the subjects with the help of parent at 15-year follow-up [18]. After quality control, the collected data were classified into 17 major food groups or 41 subgroups. Intake in g/day was calculated from the reported frequency of intake and estimated portion sizes Details on the quality control procedure and grouping can be found in a previously published article [18]. The SSD subgroup included colas, lemonades, ice-tea, sport/energy drinks, fruit squashes and nectars. For analysis purposes, SSD intake was converted into portions of 250 ml per day. In addition to SSD intake, parental education, defined as the highest level of either maternal or paternal education, was obtained from questionnaire administered to the parents.

### Clinical examination

On the day of clinical examination, study nurses welcomed the subjects and recorded their body weight (kg) and body height (cm). Following this, dental examination was performed by calibrated dentists at both the 10- and 15-year follow-ups [15, 17]. In brief, the oral cavity was illuminated using a halogen lamp (Ri-Magic, Rudolf Riester GmbH, Jungingen, Germany). The surfaces of the teeth were dried with cotton rolls to improve visibility. Caries experience was measured at both surface and tooth level using the DMF/S index according to WHO standards [19]. Non-cavitated carious lesions (NCCL/S) only in the permanent dentition were scored using criteria defined by the International Caries Detection and Assessment System (ICDAS) [20]. Using these indices, overall caries burden (DMF + NCCL/S) was calculated. Furthermore, the extent of plaque was measured visually, and binary decisions were made for each sextant for the presence of plaque. The number of plaque-affected sextants was summed from 0 (no sextant affected) to 6 (all sextants affected).

### Statistical analyses

All analyses were performed using R, version 3.3.2 (R Core Team 2016, Vienna, Austria). The intake of SSDs in portions of 250 ml per day among patients 10 and 15 years of age was defined as the exposure variable. The outcome variables were recoded binomially per surface, indicating if caries was present (0: healthy; 1: caries) at the 10- and 15-year follow-ups according to three indices: 1) DMF/S, 2) NCCL/S, and 3) DMF + NCCL/S. Parental education was classified into high (> 10 years), medium (=10 years) or low (< 10 years). Body mass index (BMI) was defined as weight in kilograms divided by height in meters squared. Following the recommendations of the working group on obesity in children and adolescents [21, 22], we classified BMI according to German age- and sex-specific percentile cut-offs for 10 and 15 year olds: (severely) underweight (<3rd percentile), normal weight (≥10th to <90th percentile); and overweight/obese (≥90th percentile). Although Kromeyer-Hauschild et al. classified BMI among children and adolescents into 5 categories, we merged them into 3 groups as the prevalence of severely underweight (<3rd percentile) and obese (>97th percentile) participants were very low in our study sample. The mode of SSD consumption was classified as not consumed, consumed with food, consumed exclusively without food, or consumed in a combination of with and without food. The energy content of SSDs was defined as diet/light/zero, normal or mixed (combination of diet/light/zero and normal). The total energy intake (kcal/day) was calculated as a continuous variable for each individual.

Simple and multiple logistic regression models were performed cross-sectionally at the 10- and 15-year follow-ups (*N* = 915 and *N* = 996, respectively), to assess the association between SSD intake and the various outcome variables. All models were adjusted for sex, parental education, age−/sex-standardized BMI categories, study cohort, plaque-affected sextants, mode of SSD consumption, energy content of SSDs, and total energy intake. As a part of sensitivity analysis, a prospective analysis was performed in which SSD intake at the 10-year follow-up was regressed on caries presence at the 15-year follow-up on all the subjects present in both follow-ups (*N* = 487). In addition to the previously adjusted covariates, prospective models were additionally adjusted for caries experience under the corresponding definition at 10 years of age. All the participants were around the same age during each follow-up; therefore, there was no variance accounted for by this variable, and the models were not adjusted for age. The adjusted odds ratios (aORs), their corresponding 95% confidence intervals (95% CIs), and *p*-values were calculated.

## Results

The mean age of the participants was 10.2 years (SD: 0.2) and 15.3 years (SD: 0.3) at the 10- and 15-year follow-ups, respectively. The study had an approximately 1:1 male-to-female ratio (Table 1). Descriptively, the overall caries burden (DMF + NCCL/S) increased from 1.81 (SD: 2.71) at the 10-year follow-up to 6.04 (SD: 8.13) at the 15-year follow-up (Table 2). During the study period, the proportion of participants affected with caries increased as well: DMF/S > 0: 15.6 to 36.1%; NCCL/S > 0: 47.9 to 66.2%; and DMF + NCCL/S > 0: 53.2 to 73.3% (Table 3). Similarly, the average SSD consumption in this study increased from 0.48 (SD: 0.85; range: 0–7.66) portions per day at the 10-year follow-up to 0.83 (SD: 1.40; range: 0–15.43) portions per day at the 15-year follow-up (Table 3). Caries-affected children tended to consume marginally more SSDs per day than caries-free children. Caries were also more frequent among males than among females.

**Table 1:** Relationship between consumption of sugar-sweetened drinks (SSDs) and relevant risk factors at the 10- and 15-year follow-ups

Variable	Category	N (%)		SSD consumption, in portions, mean (SD)	
		10-year	15-year	10-year	15-year
**Overall sample**		915 (100)	996 (100)	0.48 (0.85)**	0.83 (1.40)**
**Gender**	Male	466 (50.9)	471 (47.3)	0.57 (0.96)**	1.20 (1.72)*
	Female	449 (49.1)	525 (52.7)	0.38 (0.72)*	0.49 (0.89)*
**Parental education**	High	727 (79.4)	803 (80.7)	0.44 (0.83)**	0.72 (1.21)**
	Medium	156 (17.1)	168 (16.9)	0.62 (0.96)**	1.23 (1.88)**
	Low	31 (3.4)	24 (2.4)	0.68 (0.84)*	1.54 (2.39)*
**BMI**	Normal	735 (80.3)	850 (85.3)	0.49 (0.88)**	0.82 (1.41)**
	Underweight	114 (12.5)	62 (6.2)	0.40 (0.73)*	0.97 (1.62)*
	Overweight/obese	64 (7.0)	83 (8.3)	0.45 (0.77)	0.76 (1.11)
**Plaque-affected sextants**	0	224 (24.5)	391 (39.3)	0.44 (0.77)**	0.75 (1.23)**
	1	196 (21.4)	72 (7.2)	0.40 (0.79)	0.99 (1.58)
	2	181 (19.8)	108 (10.9)	0.45 (0.73)**	1.04 (1.42)**
	3	114 (12.5)	64 (6.4)	0.66 (1.37)	0.77 (1.34)
	4	88 (9.6)	66 (6.6)	0.59 (0.86)*	0.86 (1.78)*
	5	50 (5.5)	53 (5.3)	0.46 (0.79)	1.18 (1.83)
	6	62 (6.8)	241 (24.2)	0.43 (0.61)	1.36 (2.40)
**Mode of SSD consumption**	Not consumed	187 (20.4)	111 (11.1)	0.13 (0.49)	0.13 (0.33)
	With food	198 (21.6)	144 (14.5)	0.39 (0.80)	0.49 (1.01)
	Without food	212 (23.2)	215 (21.6)	0.33 (0.47)	0.47 (0.79)
	With/without food	318 (34.8)	526 (52.8)	0.83 (1.10)**	1.22 (1.67)**
**Energy content of SSDs**	Diet/light/zero	66 (7.2)	79 (7.9)	0.61 (1.06)	0.88 (1.20)
	Normal	212 (23.2)	205 (20.6)	0.48 (0.89)	0.92 (1.53)
	Mixed	548 (59.9)	638 (64.1)	0.60 (0.81)**	0.74 (1.13)**

Wilcoxon signed-rank test was performed to compare differences between 10- and 15-year follow-ups. * and ** indicate statistically significant difference at *p* < 0.05 and *p* < 0.01, respectively

**Table 2:** Descriptive statistics for caries experience under various definitions and relevant risk factors at the 10- and 15-year follow-ups

Variable	Category	Mean (SD)					
		DMF/S		NCCL/S		DMF + NCCL/S	
		10-year	15-year	10-year	15-year	10-year	15-year
**Overall sample**		0.36 (1.20)	1.24 (2.55)	1.45 (1.27)	4.80 (7.24)	1.81 (2.71)	6.04 (8.13)
**Gender**	Male	0.40 (1.40)	1.22 (2.32)	1.74 (2.56)	5.41 (8.43)	2.14 (2.99)	6.63 (9.21)
	Female	0.32 (0.94)	1.25 (2.75)	1.15 (1.87)	4.25 (5.94)	1.47 (2.34)	5.51 (6.99)
**Parental education**	High	0.35 (1.21)	1.18 (2.38)	1.38 (2.18)	4.66 (6.80)	1.73 (2.62)	5.84 (7.55)
	Medium	0.40 (1.16)	1.53 (3.34)	1.73 (2.63)	5.65 (9.20)	2.13 (3.12)	7.18 (10.64)
	Low	0.58 (1.06)	1.04 (1.71)	1.68 (2.30)	3.58 (5.90)	2.26 (2.49)	4.63 (6.18)
**BMI**	Normal	0.36 (1.22)	1.24 (2.55)	1.40 (2.21)	4.67 (7.07)	1.76 (2.64)	5.91 (7.96)
	Underweight	0.25 (0.88)	0.58 (1.19)	1.31 (2.32)	3.40 (5.48)	1.56 (2.72)	3.98 (5.72)
	Overweight/obese	0.59 (1.39)	1.70 (3.19)	2.30 (2.65)	7.27 (9.46)	2.89 (3.28)	8.96 (10.44)
**Plaque-affected sextants**	0	0.35 (1.58)	1.21 (2.65)	0.71 (1.24)	3.35 (5.05)	1.06 (1.99)	4.56 (6.18)
	1	0.36 (1.06)	1.35 (2.76)	1.47 (2.16)	3.96 (5.85)	1.83 (2.60)	5.31 (6.79)
	2	0.44 (1.02)	1.09 (1.69)	1.50 (2.54)	6.21 (7.47)	1.93 (3.01)	7.31 (7.67)
	3	0.40 (1.18)	1.73 (3.14)	1.68 (2.26)	4.92 (6.09)	2.08 (2.62)	6.66 (7.51)
	4	0.24 (0.76)	0.92 (1.72)	1.92 (2.53)	8.29 (8.75)	2.16 (2.82)	9.21 (9.21)
	5	0.44 (1.36)	1.07 (2.65)	2.28 (3.01)	8.02 (9.15)	2.72 (3.68)	9.09 (10.12)
	6	0.26 (0.87)	1.31 (2.66)	2.15 (2.82)	5.10 (9.06)	2.40 (2.97)	6.41 (10.12)
**Mode of SSD consumption**	Not consumed	0.39 (1.01)	0.84 (1.53)	1.48 (2.34)	3.63 (5.77)	1.87 (2.77)	4.47 (6.24)
	With food	0.46 (1.77)	1.10 (2.36)	1.26 (1.91)	4.38 (6.51)	1.73 (2.80)	5.49 (7.25)
	Without food	0.23 (0.86)	1.07 (2.04)	1.37 (2.35)	5.22 (6.63)	1.60 (2.52)	6.29 (7.30)
	With/without food	0.38 (1.03)	1.42 (2.93)	1.59 (2.37)	4.99 (7.91)	1.97 (2.74)	6.42 (8.96)
**Energy content of SSDs**	Diet/light/zero	0.17 (0.60)	1.23 (2.38)	1.73 (2.46)	5.63 (9.07)	1.89 (2.66)	6.86 (9.60)
	Normal	0.38 (0.99)	1.10 (2.30)	1.89 (2.83)	4.80 (7.13)	2.27 (3.21)	5.91 (8.02)
	Mixed	0.40 (1.37)	1.32 (2.70)	1.29 (1.99)	4.79 (7.21)	1.69 (2.51)	6.11 (8.16)

*DMF/S* decayed, missing, filled surfaces, *NCCL/S* non-cavitated carious lesions, *DMF + NCCL/S* overall caries burden (DMF/S + NCCL/S)

**Table 3:** Relationship between consumption of sugar-sweetened drinks (SSDs) and caries development under various definitions at the 10- and 15-year follow-ups

Caries definition	Category	N (%)		SSD consumption, in portions, mean (SD)	
		10-year	15-year	10-year	15-year
**Overall sample**	**–**	915 (100)	996 (100)	0.48 (0.85)^*^	0.83 (1.40)^*^
**DMF/S**	**0**	772 (84.4)	636 (63.9)	0.45 (0.75)	0.73 (1.15)^a^
	**≥1**	143 (15.6)	360 (36.1)	0.64 (1.28)	1.01 (1.74)^a^
**NCCL/S**	**0**	477 (52.1)	337 (33.8)	0.38 (0.65)^b^	0.74 (1.09)
	**≥1**	438 (47.9)	659 (66.2)	0.58 (1.02)^b^	0.87 (1.53)
**DMF + NCCL/S**	**0**	428 (46.8)	266 (26.7)	0.37 (0.63)^c^	0.73 (1.04)
	**≥1**	487 (53.2)	730 (73.3)	0.57 (1.00)^c^	0.86 (1.51)

*DMF/S* decayed, missing, filled surfaces, *NCCL/S* non-cavitated carious lesions, *DMF + NCCL/S* overall caries burden (DMF/S + NCCL/S)

^*^Statistically significant difference between 10- and 15-year SSD consumption using Wilcoxon Signed-Rank test (*p* < 0.001)

^a,b,c^Statistically significant SSD consumption between healthy and caries-affected children at 10- and 15-year follow-ups using Mann-Whitney-U test

Table 4 shows the results from the multiple logistic regression analyses performed for various definitions of caries at the 10- and 15-year follow-ups and the prospective analysis, respectively. At the 10-year follow-up, SSD intake was significantly associated with higher caries experience under DMF/S (aOR: 1.24; 95% CI: (1.04–1.48)), NCCL/S (1.33 (1.13–1.58)) and DMF + NCCL/S (1.36 (1.1–1.63)) criteria. After adjusting for all the covariates, DMF/S (1.29 (1.06–1.57)), NCCL/S (1.24 (1.03–1.49)) and DMF + NCCL/S (1.27 (1.05–1.55)) remained significantly associated with SSDs. In the 15-year follow-up, only DMF/S was found to be significantly associated with SSDs both before (1.15 (1.05–1.26)) and after (1.12 (1.01–1.25)) adjusting for the covariates. In the prospective analysis, the subset of participants for whom data were available from both follow-up time points was included (*N* = 487). There was no significant association between 10-year SSD intake and 15-year caries experience (Additional file 1: Table S1 and S2).

**Table 4:** Logistic regression analysis of the association between sugar-sweetened drink (SSD) intake (as a continuous variable) and the presence of caries. Models were adjusted for sex, parental education, age−/sex-standardized BMI categories, study cohort, plaque-affected sextants, mode of SSD consumption, energy content of SSDs, and total energy intake. The results are presented as unadjusted and adjusted odds ratios (ORs), 95% confidence intervals (95% CIs) and their corresponding *p*-values

Models	N	DMF/S		NCCL/S		DMF + NCCL/S	
		OR (95% CI)	***p***	OR (95% CI)	***p***	OR (95% CI)	***p***
**Crude associations between SSD consumption and caries**							
a.) Cross-sectional 10-year follow-up	915	**1.24** **(1.04–1.48)**	**0.02**	**1.33** **(1.13–1.58)**	**< 0.001**	**1.36** **(1.14–1.63)**	**< 0.001**
b.) Cross-sectional 15-year follow-up	996	**1.15** **(1.05–1.26)**	**0.003**	1.07 (0.97–1.19)	0.17	1.08 (0.96–1.20)	0.19
c.) Prospective analysis	487	1.19 (0.94–1.51)	0.14	1.14 (0.85–1.51)	0.38	1.06 (0.79–1.43)	0.69
**Associations between SSD consumption and caries, adjusted for covariates**							
a.) Cross-sectional 10-year follow-up	915	**1.29** **(1.06–1.57)**	**0.01**	**1.24** **(1.03–1.49)**	**0.02**	**1.27** **(1.05–1.55)**	**0.01**
b.) Cross-sectional 15-year follow-up	996	**1.12** **(1.01–1.25)**	**0.03**	1.02 (0.92–1.15)	0.68	1.03 (0.91–1.16)	0.64
c.) Prospective analysis^a^	487	1.05 (0.76–1.45)	0.79	1.07 (0.74–1.57)	0.71	1.00 (0.66–1.52)	0.99

Bold numbers indicate statistically significant associations (*p* < 0.05). ORs are adjusted for gender, plaque-affected sextants, parental education and BMI, mode of consumption, energy content of SSDs and total energy intake

*DMF/S* decayed, missing, filled surfaces, *NCCL/S* non-cavitated carious lesions, *DMF + NCCL/S* overall caries burden (DMF/S + NCCL/S)

^**a**^This model was additionally adjusted for caries experience under the corresponding definition at 10 years of age

## Discussion

This study assessed the potential associations between SSD consumption and caries experience among children from two German birth cohorts who were followed-up at 10 and 15 years of age. An association between SSD consumption and caries experience was observed at the 10-year follow-up under DMF/S, NCCL/S and DMF + NCCL/S definitions; however, in the 15-year follow-up, the association was attenuated and significant only under DMF/S criteria.

Carbohydrates on the whole [5] or SSDs [23–25] are known to be linked to the development of caries. In our study, the children in the 10-year follow-up had a low overall caries burden of 1.81. A reason for this is that the caries information was obtained only from the permanent dentition. Despite this, SSD intake in 10-year olds was found to be significantly associated with increased caries experience. Some cross-sectional studies have found associations between SSDs and caries experience in the adolescent age group [26–30]; however, most of these studies did not include NCCL/S. Although some studies reported longitudinal associations in younger children with a follow-up time ranging between 4 and 36 months [31–34], such studies were not available in an adolescent age group.

We observed a weaker association between SSDs and caries at the 15-year follow-up. The main possible explanations for this reduced association might be underreporting of SSD consumption at 15-year follow-up and improved oral hygiene habits in this age group, such as the increased use of fluoride toothpastes and mouth rinses. It has been reported that in spite of consuming more SSDs, a decrease in caries could be observed in some industrialized nations [35], which could be credited to the use of fluorides and other preventive measures [36–38]. On the other hand, the prevalence of caries still remains high in some industrialized nations [39]. Fluorides might have increased the threshold of sugar consumption to an extent [37, 40]. Another factor that might have influenced this lack of association is the effect of positive sampling that might have occurred, in which the “average” population becomes more concentrated from the loss of participants on both sides of the distribution due to a lack of interest or compliance in the research setting. Some participants might have exhibited the “Hawthorne effect”, i.e., reducing the intake of SSD consumption to fare well during the study period; alternatively, some might have underreported their SSD consumption. Although fewer participants in this study consumed a higher number of portions of SSDs, it could be seen that the caries experience was high among this population sample, which indicates a dose-response relationship, as observed in other studies [10–12]. We cannot completely rule out that a minor proportion of well-made tooth-coloured restorations might have gone undetected in the 15-year follow-up.

The main strength of the present exploratory study is that we assessed a sample size of over 900 children at both follow-up time points, ages 10 and 15 years. Our study benefitted from extensive dental data in terms of surface-level caries information and sextant-level plaque data; general health estimators, such as parental education and BMI; and dietary intake through validated FFQs. We identified some commonly observed covariates and adjusted the models for these factors. The mode of SSD consumption, the energy content of SSDs, or the total energy intake were included in the models to further reduce the influence of confounders; the inclusions of these variables did not nullify the observed associations. Considering the low proportion of remaining primary teeth in 10-year follow-up, we did not include dmf/s as one of the outcomes in our analyses. We also observed that considering only DMF/S as an index to report caries would underestimate the caries experience. In the 15-year follow-up, the mean DMF/S was only 1.24, but the mean overall caries burden was 6.04. The major component of the caries burden was contributed by the NCCL/S. Thereby; we emphasize the inclusion of NCCL/S in studies in order to estimate more accurate caries severity among populations. One main limitation we could expect in this study was the possibility of the presence of information bias, as the dietary information was obtained through questionnaires. As commonly observed in such study designs, participants may want to perform better in the study and tend to underreport some of the diet-related variables. It might be argued that adjusting for plaque-affected sextants might lead to over-adjustment and reduce the effects shown by SSDs. However, it must be noted that the extent of plaque is one of the most important aspects in the caries causal pathway, and its inclusion in the models is justified. Although the collection of dietary information in children is challenging by itself, FFQs provide a practical means of dietary assessment in large population studies, as they are associated with low participant burden and ease in data processing [41]. It might be possible that the influence of free-sugar might have influenced the results in this study; unfortunately, this information was not obtained in our study. Therefore, there might be some confounding arising from non-inclusion of this variable. However, we had adjusted for the total energy intake, which might largely counteract the effect caused by non-inclusion of free-sugar intake. Our study population was rather homogenous in terms of town of origin, age and socioeconomic status; therefore, the results from our study might not be entirely representative of children of low socioeconomic status or from other towns.

## Conclusion

The results of the present study indicate a significant positive association between SSD intake and caries, especially in 10-year-olds. The SSD consumption of 15-year-olds was significantly associated with DMF/S only. On the other hand, there was no association between SSD intake among 10-year-olds and caries incidence. The consumption and frequency of SSD intake should be reduced to a minimum or ideally avoided, especially in patients with any caries experience.

## Supplementary information

**Additional file 1: Table S1.** Descriptive statistics for caries experience among 10- and 15-year-olds (prospective data) under various definitions of caries and relevant risk factors. **Table S2.** Crude associations between consumption of sugar-sweetened drinks (SSDs) and caries development under various definitions in the sub-sample present at both 10- and 15-year follow-ups (prospective data). SSD consumption at 10-year vs. caries experience at 10- and 15-year follow-up.

## Data Availability

The datasets used and/or analysed during the current study are available from the corresponding author on reasonable request.

## Abbreviations

aOR: Adjusted odds ratio
BMI: Body mass index
CI: 95% Confidence interval
dmf/ DMF: Decayed/ missed/ filled teeth or surfaces per individuum (DMF index, WHO 1997)
FFQ: Food frequency questionnaire
GINIplus: German Infant Nutritional Intervention programme plus study
ICDAS: International Caries Detection and Assessment System
LISAplus: Lifestyle-related factors, Immune System and the development of Allergies in East and West Germany plus Study
NCCL: Non-cavitated caries lesions
OR: Odds ratio
SD: Standard deviation
SSDs: Sugar-sweetened drinks
