# Supplementary material for: Association of sugar-sweetened drinks with caries in 10- and 15-year-olds
Source: BMC Oral Health. 2020 Mar 19;20:81. doi: 10.1186/s12903-020-01068-9 (PMC7082943; doi:10.1186/s12903-020-01068-9)
Supplement: Supplementary file 1 — Additional file 1: Table S1. Descriptive statistics for caries experience among 10- and 15-year-olds (prospective data) under various definitions of caries and relevant risk factors. Table S2. Crude associations between consumption of sugar-sweetened drinks (SSDs) and caries development under various definitions in the sub-sample present at both 10- and 15-year follow-ups (prospective data). SSD consumption at 10-year vs. caries experience at 10- and 15-year follow-up. [file 12903_2020_1068_MOESM1_ESM.docx]

**Supplementary material**

**Appendix Table 1:** Descriptive statistics for caries experience among 10- and 15-year-olds (prospective data) under various definitions of caries and relevant risk factors.

**Appendix Table 2:** Crude associations between consumption of sugar-sweetened drinks (SSDs) and caries development under various definitions in the sub-sample present at both 10- and 15-year follow-ups (prospective data). SSD consumption at 10-year vs. caries experience at 10- and 15-year follow-up.

**Appendix Table 1:** Descriptive statistics for caries experience among 10- and 15-year-olds (prospective data) under various definitions of caries and relevant risk factors.

| **Variable** | **Category** | **N (%)** | | **Mean (SD)** | | | | | |
| --- | --- | --- | --- | --- | --- | --- | --- | --- | --- |
|  |  |  |  | **DMF/S** | | **NCCL/S** | | **DMF+NCCL/S** | |
|  |  | **10-year** | **15-year** | **10-year** | **15-year** | **10-year** | **15-year** | **10-year** | **15-year** |
| **Overall sample** | | 487 (100) | 487 (100) | 0.33 (1.04) | 1.19 (2.34) | 1.44 (2.32) | 5.21 (7.16) | 1.76 (2.70) | 6.40 (7.90) |
| **Gender** | Male | 232 (47.6) | 232 (47.6) | 0.39 (1.16) | 1.27 (2.35) | 1.68 (2.64) | 6.25 (8.35) | 2.07 (3.01) | 7.51 (8.94) |
|  | Female | 255 (52.4) | 255 (52.4) | 0.27 (0.92) | 1.12 (2.35) | 1.21 (1.96) | 4.27 (5.72) | 1.49 (2.36) | 5.39 (6.69) |
| **Parental education** | High | 401 (82.3) | 401 (82.3) | 0.31 (0.98) | 1.20 (2.35) | 1.38 (2.22) | 5.23 (7.17) | 1.69 (2.55) | 6.43 (7.90) |
|  | Medium | 77 (15.8) | 77 (15.8) | 0.44 (1.35) | 1.23 (2.44) | 1.64 (2.78) | 5.14 (7.00) | 2.08 (3.44) | 6.38 (7.94) |
|  | Low | 9 (1.8) | 9 (1.8) | 0.11 (0.33) | 0.33 (0.71) | 2.11 (2.20) | 5.11 (8.34) | 2.22 (2.22) | 5.44 (8.66) |
| **BMI** | Normal | 393 (80.7) | 418 (85.8) | 0.34 (1.07) | 1.17 (2.21) | 1.30 (2.17) | 4.94 (6.48) | 1.65 (2.53) | 6.11 (7.19) |
|  | Underweight | 63 (12.9) | 31 (6.4) | 0.19 (0.82) | 0.39 (0.84) | 1.71 (2.69) | 3.94 (6.77) | 1.90 (3.14) | 4.32 (7.12) |
|  | Overweight/obese | 30 (6.2) | 38 (7.8) | 0.43 (1.07) | 2.13 (3.91) | 2.63 (2.95) | 9.18 (12.03) | 3.07 (3.59) | 11.32 (12.90) |
| **Plaque-affected sextants** | 0 | 117 (24.0) | 196 (40.2) | 0.24 (0.92) | 1.22 (2.35) | 0.77 (1.37) | 3.43 (4.97) | 1.01 (1.65) | 4.66 (6.37) |
|  | 1 | 117 (24.0) | 33 (6.8) | 0.33 (1.05) | 1.24 (2.33) | 1.46 (2.18) | 4.45 (6.85) | 1.79 (2.57) | 5.70 (7.21) |
|  | 2 | 96 (19.7) | 49 (10.1) | 0.48 (1.13) | 0.90 (1.49) | 1.68 (2.79) | 6.55 (7.76) | 2.16 (3.35) | 7.45 (7.78) |
|  | 3 | 54 (11.1) | 31 (6.4) | 0.43 (1.34) | 1.61 (2.54) | 1.41 (2.21) | 5.42 (5.84) | 1.83 (2.55) | 7.03 (6.48) |
|  | 4 | 47 (9.7) | 30 (6.2) | 0.19 (0.68) | 1.23 (2.14) | 2.09 (2.59) | 10.27 (8.61) | 2.28 (2.83) | 11.50 (9.40) |
|  | 5 | 22 (4.5) | 30 (6.2) | 0.36 (1.29) | 1.37 (3.24) | 1.27 (1.98) | 8.67 (8.39) | 1.64 (3.06) | 10.03 (9.78) |
|  | 6 | 34 (7.0) | 117 (24.0) | 0.21 (0.77) | 1.09 (2.41) | 2.21 (3.27) | 5.65 (8.60) | 2.41 (3.40) | 6.74 (8.98) |
| **Mode of SSD consumption** | Not consumed | 107 (22.0) | 62 (12.7) | 0.16 (0.37) | 0.34 (0.48) | 0.45 (0.50) | 0.58 (0.50) | 0.50 (0.50) | 0.63 (0.49) |
|  | With food | 99 (20.3) | 60 (12.3) | 0.20 (0.40) | 0.20 (0.40) | 0.45 (0.50) | 0.75 (0.44) | 0.48 (0.50) | 0.77 (0.43) |
|  | Without food | 117 (24.0) | 107 (22.0) | 0.08 (0.27) | 0.37 (0.49) | 0.50 (0.50) | 0.70 (0.46) | 0.56 (0.50) | 0.79 (0.41) |
|  | With/without food | 164 (33.7) | 258 (53.0) | 0.15 (0.36) | 0.38 (0.49) | 0.49 (0.50) | 0.75 (0.43) | 0.56 (0.50) | 0.81 (0.39) |
| **Energy content of SSDs** | Diet/light/zero | 29 (6.7) | 38 (8.5) | 0.10 (0.31) | 0.26 (0.45) | 0.59 (0.50) | 0.63 (0.49) | 0.59 (0.50) | 0.66 (0.48) |
|  | Normal | 106 (24.4) | 96 (21.5) | 0.17 (0.38) | 0.39 (0.49) | 0.57 (0.50) | 0.77 (0.42) | 0.61 (0.49) | 0.81 (0.39) |
|  | Mixed | 299 (68.9) | 313 (70.0) | 0.15 (0.36) | 0.36 (0.48) | 0.45 (0.50) | 0.72 (0.45) | 0.53 (0.50) | 0.80 (0.40) |

**Appendix Table 2:** Crude associations between consumption of sugar-sweetened drinks (SSDs) and caries development under various definitions in the sub-sample present at both 10- and 15-year follow-ups (prospective data). SSD consumption at 10-year vs. caries experience at 10- and 15-year follow-up.

| **Caries definition** | **Category** | **N (%)** | | **SSD consumption,**  **in portions,**  **mean (SD)** | |
| --- | --- | --- | --- | --- | --- |
|  |  | **10-year** | **15-year** | **10-year** | **15-year** |
| **Overall sample** | **-** | 487 (100) | 487 (100) | 0.40 (0.77)^*^ | 0.81 (1.30)^*^ |
| **DMF/S** | **0** | 416 (85.4) | 315 (64.7) | 0.37 (0.64)^a^ | 0.36 (0.61) |
|  | **≥1** | 71 (14.6) | 172 (35.3) | 0.60 (1.29)^a^ | 0.47 (1.00) |
| **NCCL/S** | **0** | 255 (52.4) | 138 (28.3) | 0.30 (0.53)^b^ | 0.35 (0.56) |
|  | **≥1** | 232 (47.6) | 349 (71.7) | 0.51 (0.96)^b^ | 0.42 (0.84) |
| **DMF+NCCL/S** | **0** | 228 (46.8) | 108 (22.2) | 0.30 (0.54)^c^ | 0.37 (0.59) |
|  | **≥1** | 259 (53.2) | 379 (77.8) | 0.49 (0.92)^c^ | 0.41 (0.82) |
| ^*^ Statistically significant difference between 10- and 15-year SSD consumption using Wilcoxon Signed-Rank test (p<0.001)  ^a,b,c^ Statistically significant SSD consumption between healthy and caries-affected children at 10- and 15-year follow-ups using Mann-Whitney-U test | | | | | |
